# NNTox: Gene Ontology-Based Protein Toxicity Prediction Using Neural Network

**DOI:** 10.1038/s41598-019-54405-6

**Published:** 2019-11-29

**Authors:** Aashish Jain, Daisuke Kihara

**Affiliations:** 1 Department of Computer Science, Purdue University, West Lafayette, IN 47907 USA; 2 Department of Biological Sciences, Purdue University, West Lafayette, IN 47907 USA; 3 Department of Pediatrics, University of Cincinnati, Cincinnati, OH 45229 USA

**Keywords:** Protein function predictions, Sequence annotation

## Abstract

With advancements in synthetic biology, the cost and the time needed for designing and synthesizing customized gene products have been steadily decreasing. Many research laboratories in academia as well as industry routinely create genetically engineered proteins as a part of their research activities. However, manipulation of protein sequences could result in unintentional production of toxic proteins. Therefore, being able to identify the toxicity of a protein before the synthesis would reduce the risk of potential hazards. Existing methods are too specific, which limits their application. Here, we extended general function prediction methods for predicting the toxicity of proteins. Protein function prediction methods have been actively studied in the bioinformatics community and have shown significant improvement over the last decade. We have previously developed successful function prediction methods, which were shown to be among top-performing methods in the community-wide functional annotation experiment, CAFA. Based on our function prediction method, we developed a neural network model, named NNTox, which uses predicted GO terms for a target protein to further predict the possibility of the protein being toxic. We have also developed a multi-label model, which can predict the specific toxicity type of the query sequence. Together, this work analyses the relationship between GO terms and protein toxicity and builds predictor models of protein toxicity.

## Introduction

Proteins carry out various functions in a cell, forming functional networks and signaling pathways that are essential to sustain life. Understanding the function of component proteins in the networks is a fundamental step to obtain critical insights into complex cellular mechanisms. As a means to elucidate the function of a protein and the relationship between the function and the sequence or the structure of the protein, experimentally, it is common to construct mutants of the protein and test their function *in vitro* and *in vivo*. Advancements in synthetic biology^1,2^ as well as protein design^3^ have made it now possible to construct artificial proteins that fold and assemble into desired structures and achieve specific tasks in a cell. Artificial protein synthesis has also revolutionized the biotechnology industry, where the technique has been used to program microbes to produce drugs at reduced production cost, to create disease-resistant crops that improve the yield, or to design new vaccines and therapeutic antibodies to cure diseases^4–6^.

While there are many applications of constructing desired artificial peptides and proteins, a potential problem is the production of harmful or toxic proteins. There are two scenarios where toxic proteins may be constructed: One situation would be that a newly designed protein happens to have an unexpected harmful function. There are many aspects of cell function that are still unclear, thus, foreseeing such side effects when designing a new protein may be very difficult. The second possible case would be an intentional design or release of toxic proteins for biological attack^7^. To prevent release of toxic proteins, there are ongoing efforts to build systems and devices that collect unknown proteins or organisms together that identify proteins with potential harm^8–11^. There is a strong demand for such systems for lab facilities of gene synthesis, places where many people gather, e.g. airports, and war zones where biological attack might occur.

A computational algorithm for detecting toxic proteins should take a protein or DNA sequence as input and alerts if the protein can be harmful. ThreatSEQ developed by Battelle Memorial Institute identifies sequences of concern by comparing them with a curated database of known toxic proteins^12^. ToxinPred^13^ and other series of methods developed by the Raghava group target detection of toxic bacterial peptides using machine learning methods based on sequence information^14,15^. ClanTox uses a machine learning method that was trained on known peptide ion-channel inhibitors^16^. These methods are similar in approach in that they use sequence information. Moreover, the methods except for ThreatSEQ have a limited application to peptide toxins.

In this paper, we present a new method, NNTox (Neural Network-based protein Toxicity prediction), which can predict the toxicity of a query protein sequence based on the protein’s Gene Ontology (GO) annotation^17^. GO is a controlled vocabulary of function of proteins and has been widely used for function annotation and prediction. Previously, our lab has developed a series of function prediction methods^18,19^ including PFP^20–22^ and Phylo-PFP^19^, which have been shown to be among the top-performing function prediction methods in the community-wide automatic function prediction experiment, Critical Assessment of protein Function Annotation (CAFA)^23,24^. Here, we show that the toxicity of proteins can be well predicted from GO terms that are predicted by PFP. First, we examined the distribution of GO terms in annotations of toxic proteins and showed that GO terms are promising features for predicting toxicity. Next, we developed a neural network for predicting protein’s toxicity from their GO term annotations. Finally, we further extended the method to predict the mode of action of toxicity of a protein.

## Methods

First, we will describe the datasets used in this study. Then, we explain the neural network model of NNTox.

### Toxic protein dataset

Toxin proteins were collected from the UniProtKB-SwissProt database^25^ using the keyword “Toxin” (UniProtKB KW-0800). A total of 6,497 toxin proteins were obtained. From the 6,497 toxin proteins, we collected a set of 1,506 unique GO terms that were included in their GO annotations. The GO term of “toxin activity” (GO:0090729) was removed from the collection because this term obviously related to toxicity and can bias prediction if it is included in the annotation of proteins in the training and testing set for the toxicity prediction. From this toxin protein set, we removed proteins that were redundant to other proteins in terms of their GO term annotations. We did not use sequence similarity for the redundancy criterion because the input to our model is GO terms. The non-redundant dataset contained 488 toxin proteins.

Non-toxin proteins were also collected from UniProtKB SwissProt using the following two conditions: (1), they are not tagged with the keyword “Toxin”. (2), 95% of GO terms annotating the protein belong to the toxin GO term set. The second criterion makes most of the GO term annotation of toxin and non-toxin proteins very similar. Using this approach 82,583 non-toxin proteins were obtained. Then, as was done for the toxin protein dataset, proteins with redundant GO annotations were removed, which resulted in 6,594 non-toxin GO proteins.

The Toxin keyword had 11 sub-classes, which were cardiotoxin (134/8), enterotoxin (94/12), neurotoxin (2744/100), ion channel impairing toxin (2429/74), myotoxin (121/22), dermonecrotic toxin (148/4), hemostasis impairing toxin (865/95), G-protein coupled receptor impairing toxin (186/33), complement system impairing toxin (160/6), cell adhesion impairing toxin (207/18), and viral exotoxin (9/4). The first number in the parentheses is the total number of proteins in the sub-class downloaded from UniProtKB-SwissProt while the second number is those in the non-redundant toxin proteins. Using this information, we compiled a dataset of the mode of action of the toxin proteins. Out of the 488 non-redundant toxin proteins, 270 proteins had information of the mode of action. A protein is assigned to multiple classes if it belongs to more than one sub-class keywords. Out of the 270 proteins, 173 proteins belong only to one sub-class, 88 proteins have two assigned sub-classes, and 9 have three sub-classes.

### Feature vector representing a protein

A protein in the dataset is represented by a vector of 2,596 binary (1 or 0) values (except for the last position), which indicates existence of the particular GO term in its GO annotation. 2595 GO terms represents all the GO terms found in toxin proteins as well as general GO terms that frequently appear in UniProtKB database (concretely, all GO terms that annotate more than 1000 proteins). The last position of the vector represents the number of GO terms that are associated with the protein but are not present among the above 2,595 GO terms. Using only toxin GO terms in the feature vector limits the scope of GO terms that the network can see and using all (>35,000) will lead to spare features. As a middle ground, we added top background GO term in the feature vector as well.

### Neural network models

**Figure 1:**
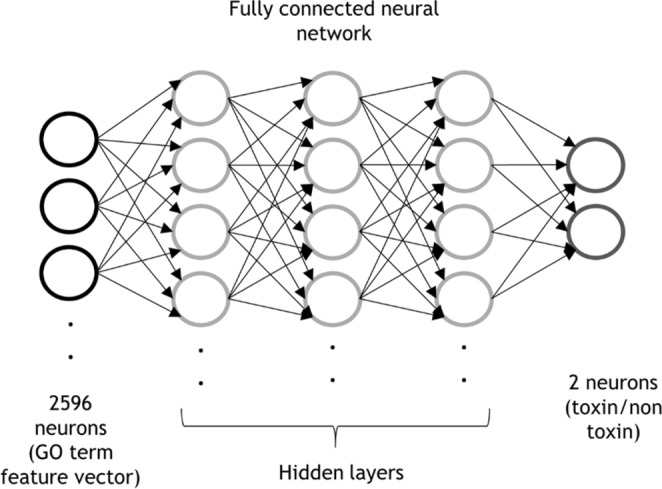
The network architecture of NNTox for toxin/non-toxin binary prediction.

We used a five-layer fully connected feedforward neural network for the toxin/non-toxin prediction (Fig. 1). The input layer has 2,596 neurons representing the GO term feature vector. The input layer is connected to three hidden layers, each of which has 200 neurons. The last layer uses the softmax nonlinearity to convert the output into class probability, toxin and non-toxin. Neurons are connected with a sigmoidal activation function. The code is available at http://www.github.com/kiharalab/NNTox.

Predicting the mode of action of toxin proteins is a multi-label classification problem, where one toxin could have more than one mode of action. For example, conotoxin, a snail toxin, is both a neuro-toxin and an ion channel inhibitor toxin. Thus, classes are not mutually exclusive. We modified the neural network described above to perform multi-label prediction, by replacing softmax in the last layer with computing the sigmoid cross-entropy loss. In the sigmoid cross-entropy loss, the loss calculated for every label is independent of the loss in other labels, and thus allows for multiple labels to be predicted.

The sub-classes of toxins are imbalanced, e.g. neurotoxin and ion channel inhibiting toxin have more proteins than other sub-classes. This can cause bias in the network while training towards highly represented classes. To overcome this problem, we added a weight to each correct class prediction in the multi-label neural network, where the weight is inversely proportional to the number of the times that class is present in the training set. For a protein, v = [v_1_, v_2_, … v_11_] is the label vector, where v_i_ = 1 represents that the protein has the mode of action i. For each mode of action i, we calculated the positive count (i), i.e., the number of times v_i_ = 1 and the negative count (i), i.e., the number of times v_i_ = 0 in the training dataset. The weight w_i_ given to a mode of action/class i is w_i_ = (negative count (i))/(positive count (i)). Thus, the weight is 1 if the number of positive and negative counts is equal while giving more weight as the positive count decreases.

### Training and validation with nested cross-validation

Training was performed with backpropagation using the ADAM optimizer, implemented in TensorFlow^26^ We performed a five-fold nested cross validation to tune four hyper-parameters: the number of neurons in hidden layer [10, 50, 100, 200, 500], the regularization strength [10, 1, 0.1, 0.01, 0.001], the learning rate [10, 1, 0.1, 0.01, 0.001] and the number of epochs [100, 500, 1000, 2000, 5000]. Shown in the parentheses are the values tested for each hyper-parameter.

Nested cross-validation provides robust and unbiased training and testing using the full data available from the dataset. In the nested cross-validation there were two cross validation loops. In the outer loop, the dataset was divided into k (=5) subsets, where one subset was considered as the test set and the rest are used for training & validation set, and the test set was changed for k times. Furthermore, the inner loop was to perform a cross-validation on the training & validation set, i.e. the set was divided into k (=5) pieces again and one of them was considered as the validation set. Each different combinations of hyper-parameters were trained on the training set and tested on the validation set. This was performed for k times by changing the validation set. Then, the best hyper-parameter was chosen based on the average error on the k validation set, and the model trained using the hyper-parameter set on all training and validation set was applied to the testing set. This is repeated for k times, and the final result was the average performance on the k test sets.

### Protein function prediction with PFP

We examined the performance of NNTox using two sets of GO terms for proteins. First, we tested NNTox using the GO annotations of proteins obtained from UniProtKB-SwissProt. This is to test the performance of the architecture of NNTox in the best possible cases when all the correct GO terms are known. Second, we used a GO-term prediction method, PFP, to predict GO terms of each protein and trained NNTox on the predicted GO terms. This is to simulate the situation when true GO terms for a query protein are not present.

PFP was developed in our group and has been successful in the Critical Assessment of protein Function Annotation algorithms (CAFA). PFP uses PSI-BLAST^27^ to retrieve similar sequences from a database to a query sequence and obtains GO-term annotations from the sequences with an E-value of up to 125. Then, each GO term will be assigned with a score that reflects the E-value of sequences that have the GO term in their annotation as well as the conditional probability that the GO term occurs given other GO terms are observed. For the sequence database, we used UniProtKB Swiss-Prot downloaded in March 2018. To avoid retrieving GO terms from the query protein itself, sequences retrieved with an E-value of 0 were discarded.

PFP provides a confidence score to each GO term predicted that ranges from 0.0 to 1.0 with 1.0 for the highest confidence (Supplementary Table S1). Using PFP, we devised a simple baseline strategy to predict if a protein is toxin or not directly from assigned GO terms. If PFP predictions include the “toxin activity” GO term (GO:0090729) with high confidence (>=0.9) then we label the protein as a toxin. We also trained NNTox network with PFP-predicted GO terms. Only predicted terms were used for this training, i.e. known GO term annotations were not considered to simulate the situation that query proteins do not have any known annotations. We removed the “toxin activity” GO term from the PFP predictions as having this GO term would bias the model and make the toxin prediction easy.

### Additional baseline method

To evaluate the performance of NNTox, we developed a naïve GO term based baseline approach. In this approach, a protein is classified as toxin if all the GO terms associated with it are present in the Toxin GO term set. This approach reflects the idea that if a set of GO terms are already known to be associated with a toxin, we classify a new protein associated with those GO terms as toxin as well. For baseline method, the non-redundant toxin protein dataset was split into a 70:30 train:test ratio, where 70% of the dataset was used to create the Toxin GO term set. The method was tested with 30% of the toxin test dataset and all the non-redundant non-toxin proteins.

### Prediction evaluation

Prediction accuracy was evaluated with the F1 score. The precision P, recall R and F1 score was calculated as

$$\begin{array}{rcl}P & = & \frac{TP}{TP+FP}\\ R & = & \frac{TP}{TP+FN}\\ F1 & = & \frac{2\,\ast \,P\ast \,R}{(P+R)}\end{array}$$

where TP is the total number of proteins that are toxin and were predicted correctly as toxin, FP is the total number of proteins that are non-toxin but predicted as toxin, and FN is the total number of proteins which are toxin but predicted as non-toxin.

## Results

### GO term specificity for toxin proteins

To begin with, we examined if any GO terms have a specific association with the toxicity of proteins. We computed the specificity of GO terms for toxin proteins, which was defined as the fraction of the toxin proteins that are annotated with the specific GO term among all proteins in UniProtKB-SwissProt with the GO term annotation. Table 1 lists top 20 GO terms with the highest toxin specificity. Supplementary Table S2 provides a complete list of GO terms associated with toxin keywords. Besides GO terms that are apparently related to toxins, e.g. those with the word “inhibitor” in their description, there are highly toxin-specific terms that do not directly indicate toxicity.

**Table 1:** Toxin specific GO terms.

GO ID	Function	Toxin Spec. (%)^a^
0035792	other organism postsynaptic membrane	100.00 (554)
0072556	other organism presynaptic membrane	98.14 (317)
0042151	nematocyst	91.64 (252)
0030550	acetylcholine receptor inhibitor activity	91.11 (123)
0019871	sodium channel inhibitor activity	89.89 (169)
0008200	ion channel inhibitor activity	87.89 (1415)
0016248	channel inhibitor activity	87.56 (1415)
0099602	neurotransmitter receptor regulator activity	75.46 (123)
0034548	acetylcholine receptor regulator activity	75.46 (123)
0070290	N-APE-PLD D activity^b^	75.35 (214)
0004630	phospholipase D activity	75.09 (214)
0016247	channel regulator activity	71.72 (1415)
0030547	receptor inhibitor activity	69.44 (125)
0009405	pathogenesis	66.26 (6497)
0102568	phospholipase A2 activity (12-DOPE)^c^	59.51 (319)
0102567	phospholipase A2 activity (12- DPPtdCho)^d^	59.51 (319)
1903963	arachidonate transport	59.48 (342)
0050482	arachidonic acid secretion	59.47 (342)
0017080	sodium channel regulator activity	59.31 (172)
0004623	phospholipase A2 activity	58.41 (375)

^a^The number of toxin proteins with the GO term is shown in the parenthesis. ^b^N-acylphosphatidylethanolamine-specific phospholipase D activity. ^c^Phospho-lipase A2 activity consuming 12-dioleoylphosphatidylethanolamine. ^d^Phospho-lipase A2 activity (consuming 12-dipalmitoylphosphatidylcholine).

The first GO term in the table, “Other organism postsynaptic membrane” (GO: 0035792) has 100% of the toxin specificity. Proteins with this GO term are indeed toxins, e.g. alpha-conotoxin in a sea snail (Uni-Prot ID: CDKA_CONVX) and cobrotoxin in Chinese cobra (UniProt ID: 3S1CB_NAJAT). These toxins bind to nicotinic acetylcholine receptors, inhibiting them, and impairing neuromuscular transmission. Thus, it is involved in neurotoxicity and ion channel impairing toxicity. “N-acylphosphatidylethanolamine-specific phospholipase D (NAPE-PLD) activity” (GO: 0070290, example proteins: UniProt ID: A1lB1_LOXIN) has a high toxin specificity of 75.35%. Phospholipid D catalyzes the hydrolysis of sphingomyelin and induces complement-dependent hemolysis, dermonecrosis, blood vessel permeability, and platelet aggregation. Thus, it is involved in dermonecrotic and complement system toxicity. It is possessed by recluse spiders and causes necrotic damage. “Phospholipase A2 activity” (GO:0004623), the last one in the table, has a toxin specificity of 58.41% with neurotoxin specificity of 22%, myotoxin specificity of 14%, and hemostasis impairing toxin specificity of 23%. Phospholipase A2 catalyzes the calcium-dependent hydrolysis of the 2-acyl groups in 3-sn-phosphoglycerides. It affects neuromuscular transmission by blocking acetylcholine release from the nerve termini. It also has anticoagulant activity and weakly inhibits ADP-induced platelet aggregation. The protein with this activity exists in venomous snakes, e.g. Chinese krait (UniProt ID: PA2B1_BUNMU) and Nikolsky’s Viper (UniProt ID: PA2B2_VIPBN). Overall the results show GO terms are promising features for predicting protein toxicity.

### Performance of toxin prediction

In this section we discuss the performance of our NNTox on distinguishing toxin and non-toxin proteins. We compare the performance with the baseline methods. Table 2 summarizes the results. The table shows precision, recall, and the F1 score, which was defined as the harmonic mean of precision and recall of toxin protein prediction.

**Table 2:** Summary of the toxin prediction.

Method	Precision	Recall	F1 score
**With GO annotation**			
Baseline exact	0.029	0.626	0.055
Baseline 1 mismatch	0.023	0.714	0.044
Baseline 2 mismatches	0.021	0.769	0.041
NNTox (GO Annotation)	0.903	0.898	0.900
**With PFP prediction**			
Baseline exact	0.110	0.156	0.129
Baseline 1 mismatch	0.102	0.184	0.131
Baseline 2 mismatches	0.115	0.259	0.159
PFP	0.873	0.535	0.663
NNTox (PFP)	0.801	0.750	0.775
PFP + NNTox(PFP)	0.807	0.781	0.794

**Figure 2:**
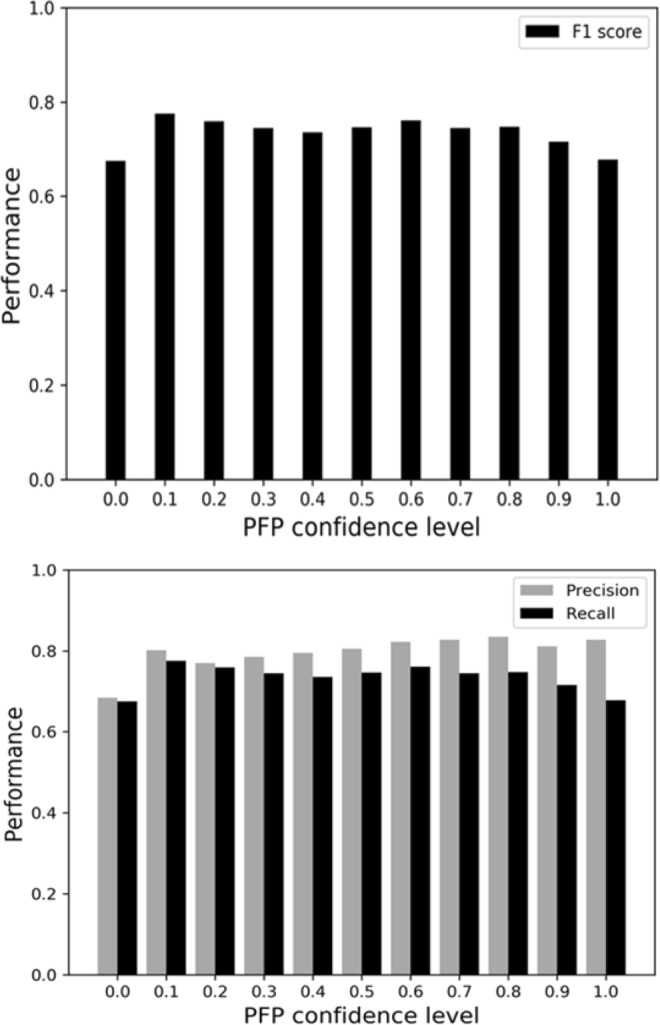
F1 score, precision, and recall of toxin prediction for different PFP’s GO prediction confidence levels.

The baseline method is explained in Methods. NNTox (GO Annotation) used the GO annotations of proteins from UniProtKB-SwissProt. “PFP” checked if the “toxin activity” GO term was predicted with 0.9 or a higher confidence score. NNTox (PFP) uses predicted GO terms by PFP using 0.1 as the prediction confidence cutoff value (Fig. 2). PFP + NNTox(PFP) is a two-step prediction using first PFP and then to apply NNTox(PFP) for proteins that are not identified as toxin by PFP.

In the first three rows of Table 2, we showed the prediction performance one can obtain by simply comparing GO annotation of a target protein with known proteins in the reference database (the baseline method). When the exact match of GO terms was counted, recall for toxin proteins was 0.626. When the condition was relaxed, allowing 1 or 2 miss matches of GO terms, the recall for toxin proteins naturally increased to 0.714 by sacrificing the precision. This is intuitive because with 1 mismatch allowed, proteins which had only one GO term not present in the toxin GO set were now predicted as toxins as well but with the cost of false positives. F1 scores of the baseline method were as low as 0.055 due to low precision values that were caused by a large number of false positives (i.e. non-toxin proteins predicted as toxins).

In contrast, prediction by NNTox performed substantially better than the baseline method. The precision and recall for detecting toxin proteins was 0.903 and 0.898, respectively, indicating that the predictions made for toxin and non-toxin proteins were well balanced. The NNTox F1 score was 0.900, which is a clear contrast compared to baseline method that showed substantially lower F1 score.

The second half of Table 2 shows results using PFP predicted GO terms. Using predicted GO terms, the baseline method showed lower recall as compared with results using GO annotations. This is because predicted GO terms for a protein have a low random chance to perfectly agree with toxin GO terms. As another baseline, PFP prediction was also directly used to determine if a protein is toxin by checking if the prediction included “toxin activity” GO term with a high confidence (>=0.9). This approach performed better than the baseline method showing an F1 score of 0.663 and a recall of 0.535. Thus, about half of the toxin proteins were identified correctly by the PFP baseline. NNTox performed better than the baseline methods and the PFP baseline with an F1 score of 0.775, although the performance was worse than the cases with correct GO annotation. For NNTox, we used predicted GO terms with PFP’s confidence score of over 0.1, since that gave the best performance (Fig. 2). We also tested a two-step prediction process where PFP and NNTox with PFP predicted GO terms were combined (the last row in Table 2). First, the protein was determined to be toxin based on direct PFP predictions. Then, if the protein is not predicted to be toxin, then NNTox was applied. This procedure further improved NNTox in all the evaluation metrics. The F1 score increased from 0.775 to 0.794. Looking closely, the first step of the PFP application filtered 261 toxin proteins correctly (i.e. true positives), then additional 120 toxin proteins were selected by the NNTox.

**Figure 3:**
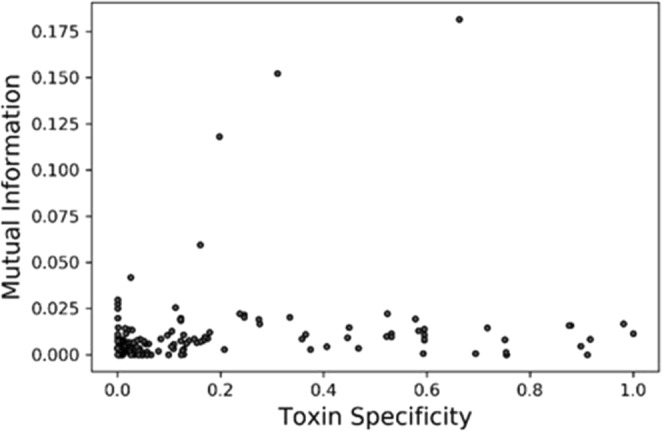
Mutual information and toxin specificity of GO terms for toxin/non-toxin classification.

In Fig. 3, we analyzed the importance of each GO term in distinguishing toxin and non-toxin proteins. For each GO term in the feature vector, we computed the mutual information relative to the toxin classification. As shown, a large specificity of a GO term does not necessarily mean a large mutual information for the classification. Such cases happen for GO terms that are highly specific for toxins but only appear in annotation of a small number of proteins, thus not much helpful for the classification for many proteins in the dataset. The top three GO terms were pathogenesis (GO:0009405), interspecies interaction between organisms (GO:0044419) and multi-organism process (GO:0051704), which is not surprising as these terms highly indicative of a protein being toxin.

### Neural network visualization

**Figure 4:**
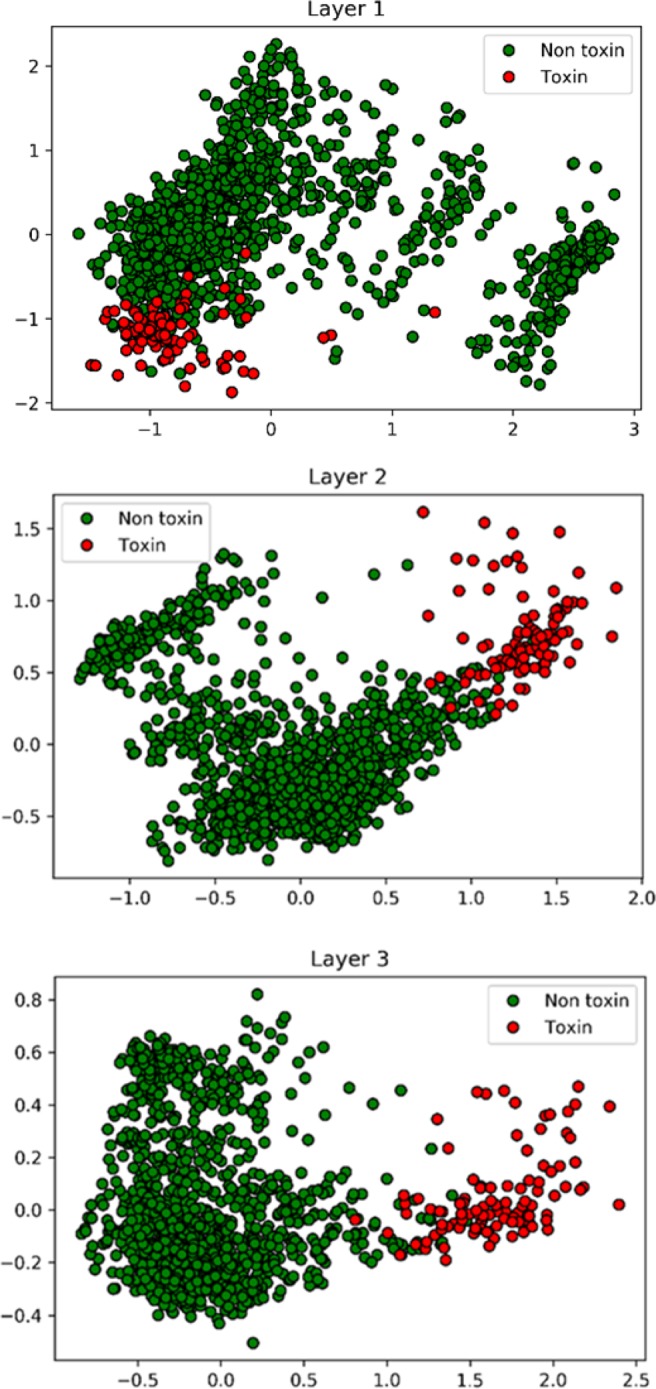
Separations of toxin and non-toxin proteins in the neural network layers.

In Fig. 4 we visualized the network to illustrate how the neural network model separated the toxin and non-toxin proteins using the principal component analysis (PCA). For each protein in the non-redundant tox-in/non-toxin set, we ran the trained network and calculated the output of each of the three hidden layers and passed it through the sigmoid activation function. The top figure shows that toxin proteins (red) mostly overlapped with non-toxin proteins in the PCA space. The distinction between the two classes became substantially clearer in the second layer (the middle panel), and further improved in the third layer. Thus, as the network went deeper and the model complexity increased, the model was able to separate the two classes better.

Outputs from each of the three hidden layers of the neural network for toxin (red) and non-toxin (green) proteins are visualized by PCA. The x- and the y-axis are the first and the second principal components of the output values of the layer through the sigmoid activation function.

### Prediction of toxin mode action

Next, we developed a multi-label neural network model, which predicts the mode of action of a toxin protein. The input to the model is the same feature vector of GO terms and the output is a binary vector for the 11 modes of action. Multiple action predictions are also allowed for a protein, which makes the prediction task more complex. To evaluate the prediction performance of the model, we computed the elementwise accuracy of the predicted vector (Table 3) as usually used for multi-label classification^28^, where the number of correctly predicted modes for each of the target proteins was counted. NNTox (Mode of action) showed good performance with an accuracy of over 0.8, even when predicted GO terms were used. The high accuracy indicates that the method was overall successful in not only for pointing out the correct mode of the toxin proteins but also in avoiding over predicting incorrect modes.

**Table 3:** Summary of the mode of action prediction accuracy.

Input GO terms	Accuracy
UniProtKB	0.879
Prediction by PFP	0.825

The values are the average for test sets in the five-fold nested cross-validation. In this multi-class prediction, a prediction output for a protein is a binary vector of 11 values, where 1 indicates the class is predicted and 0 for a negative prediction for the class. The accuracy was computed by counting the agreement of the predicted binary class for each toxin mode of action in all the proteins.

11 modes shown on the x-axis are: C, cardiotoxin; EN, enterotoxin; N, neurotoxin; IC, ion channel impairing toxin; M, myotoxin; D, dermonecrotic toxin; H, hemostasis impairing toxin; GCR, G-protein coupled receptor impairing toxin; CS, complement system impairing toxin; CA, cell adhesion impairing toxin; V, viral toxin. In the parentheses, the number of proteins of the mode is shown. 173 toxin proteins that have only one mode of action were analyzed. Black bars, predictions using GO annotations from UniProtKB; gray bars, predictions using PFP’s GO term predictions.

**Figure 5:**
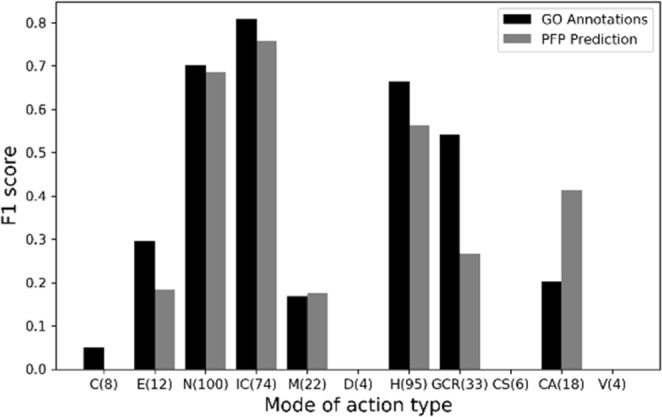
F1 scores of single-mode toxin proteins of 11different modes of action.

Figure 5 shows the F1 score of each mode of action separately for toxin proteins with a single action mode. Precision and recall values are provided in Supplementary Tables S3 and S4. Naturally, F1 scores correlated strongly with the number of data available for modes, which is shown in the parentheses of the mode labels on the x-axis. A relatively high F1 score was observed for modes that have more data, but low scores were resulted in for modes with small data size. Thus, the data availability of the current database limits the prediction performance for several toxin modes, nevertheless, the results indicate that in principle the model is reasonable and will only improve by the increase of toxin data to be available in the future.

Among the toxin protein dataset with the mode of action, there are 88 proteins that have two mode labels. Here we examine predictions made to the two largest toxin groups with two labels. 54 out of 88 proteins are labeled as neurotoxin (N) and Ion channel impairing toxin (IC). Out of them, 30 (55.6%) had the exactly correct predictions, i.e. correct positive predictions for the two labels and correct negative predictions to the other modes. For 9 other cases, the two labels, N and IC, were correctly predicted but with other false positive predictions. Finally, 48 of them (88.9%) had at least 1 mode, either N or IC, correctly predicted. The second-largest group with two modes were with hemostasis impairing toxin (H) and cell adhesion impairing toxin (CA), with 16 proteins. For this group, five of them have the exact correct prediction, and another protein was counted if we include the prediction with the two correct modes and one more over-predicted mode (37.5%). The number of proteins with at least one correctly predicted mode, H or CA, was 12 (75.0%). Thus, overall, NNTox (Mode of action) was able to capture the dual labels of the proteins reasonably well.

## Discussion

Here, we developed NNTox, which predicts the toxicity of proteins via GO term annotation. In contrast to existing methods that compare a query protein sequence to known toxin proteins, NNTox’s approach is less dependent on the known similar toxin proteins because prediction is made via GO terms. This approach exploits the success of general function predictors that have constantly been improving in the past years. We used PFP for the current development because it was developed by our lab and is one of the top-performing methods in the field. As the function prediction method improves, the toxin prediction by NNTox will also improve. Performance is also expected to improve by using additional input features, such as protein local structure information, e.g. protein main-chain conformation^29^, which can be predicted with a stable accuracy.

The multi-label classification performed for toxin action mode prediction showed high elementwise accuracy (Table 3). Naturally, the accuracy for each mode was correlated to the data size of the category, which indicates that the architecture of the model is appropriate for this task and will further improve as more data become available.

In this work, we trained the network model so that the overall F1 score was maximized. The method can also be trained differently, for example, in a way to increase the sensitivity of toxin detection (allowing more false positives), considering that missing life-threatening toxins can cause a catastrophic outcome.

## Supplementary information

Supplementary Table S1, S3, S4

Supplementary Table S2

## Data Availability

The code and the dataset used in this study are made available at http://www.github.com/kiharalab/NNTox and http://kiharalab.org/nntox_dataset/.
