# Supplementary material for: NNTox: Gene Ontology-Based Protein Toxicity Prediction Using Neural Network
Source: Sci Rep. 2019 Nov 29;9:17923. doi: 10.1038/s41598-019-54405-6 (PMC6884647; doi:10.1038/s41598-019-54405-6)
Supplement: Supplementary file 1 — Supplementary Table S1, S3, S4 [file 41598_2019_54405_MOESM1_ESM.docx]

*Supplementary Information for*

**NNTox: Gene Ontology-Based Protein Toxicity Prediction Using Neural Network**

Aashish Jain & Daisuke Kihara*

Department of Computer Science, Department of Biological Sciences

Purdue University

*Contact: dkihara@purdue.edu

**Supplementary Table S1.** F1 score of GO term prediction by PFP for the non-redundant toxin dataset (488 toxin proteins).

| PFP confidence cut-off | Precision | Recall | F1 score |
| --- | --- | --- | --- |
| 0.0 | 0.282 | 0.562 | 0.376 |
| 0.1 | 0.602 | 0.522 | 0.560 |
| 0.2 | 0.699 | 0.497 | 0.580 |
| 0.3 | 0.741 | 0.476 | 0.580 |
| 0.4 | 0.765 | 0.459 | 0.575 |
| 0.5 | 0.786 | 0.446 | 0.570 |
| 0.6 | 0.806 | 0.436 | 0.566 |
| 0.7 | 0.821 | 0.410 | 0.547 |
| 0.8 | 0.831 | 0.393 | 0.533 |
| 0.9 | 0.849 | 0.361 | 0.507 |
| 1.0 | 0.860 | 0.293 | 0.436 |

A protein was considered as toxin if the GO term “toxin activity” (GO:0090729) was included among predicted terms with the confidence above the cutoff used.

**Supplementary Table S2.** Association of GO terms with Toxin Keywords in UniProt.

This Table is provided in a separate Excel file. The file shows the toxin specificity, i.e. how much GO terms associate with toxin keywords of UniProt. The first and the second columns are the ID and the text description of GO terms, the toxin specificity (the third column) shows the fraction of proteins in UniProtKB-SwissProt that are toxins (i.e. with a keyword ‘Toxin’ UniProtKB KW-0800) among all the proteins in UniProtKB-SwissProt. The rest of the columns, Toxin Mode 1 to 3 show the dominant action mode(s) of the toxin if any that share above 10% of the toxin proteins. The labels of the modes of toxin are:

C: Cardiotoxin

EN: Enterotoxin

N: Neurotoxin

IC: Ion channel impairing toxin

M: Myotoxin

D: Dermonecrotic toxin

H: Hemostasis impairing toxin

GCR: G-protein coupled receptor impairing toxin

CS: Complement system impairing toxin

CA: Cell adhesion impairing toxin

V: Viral exotoxin

**Supplementary Table S3.** Results of the mode of action prediction for individual categories using UniProtKB GO annotations.

| Mode of Action | Precision | Recall | F1 score | Total Number of Proteins |
| --- | --- | --- | --- | --- |
| Cardiotoxin | 0.031 | 0.125 | 0.050 | 8 |
| Enterotoxin | 0.267 | 0.334 | 0.296 | 12 |
| Neurotoxin | 0.736 | 0.670 | 0.702 | 100 |
| Ion channel impairing toxin | 0.819 | 0.797 | 0.808 | 74 |
| Myotoxin | 0.135 | 0.227 | 0.169 | 22 |
| Dermonecrotic toxin | 0 | 0 | 0 | 4 |
| Hemostasis impairing toxin | 0.774 | 0.579 | 0.663 | 95 |
| G-protein coupled receptor impairing toxin | 0.413 | 0.788 | 0.542 | 33 |
| Complement system impairing toxin | 0 | 0 | 0 | 6 |
| Cell adhesion impairing toxin | 0.146 | 0.334 | 0.203 | 18 |
| Viral exotoxin | 0 | 0 | 0 | 4 |

Figure 5 shows the F1 scores taken from Table S3 and S4.

**Supplementary Table S4.** Results of the mode of action prediction for individual categories using PFP predictions.

| Mode of Action | Precision | Recall | F1 score | Total Number of Proteins |
| --- | --- | --- | --- | --- |
| Cardiotoxin | 0 | 0 | 0 | 8 |
| Enterotoxin | 0.109 | 0.583 | 0.184 | 12 |
| Neurotoxin | 0.630 | 0.750 | 0.685 | 100 |
| Ion channel impairing toxin | 0.674 | 0.865 | 0.757 | 74 |
| Myotoxin | 0.109 | 0.455 | 0.175 | 22 |
| Dermonecrotic toxin | 0 | 0 | 0 | 4 |
| Hemostasis impairing toxin | 0.721 | 0.463 | 0.564 | 95 |
| G-protein coupled receptor impairing toxin | 0.184 | 0.485 | 0.267 | 33 |
| Complement system impairing toxin | 0 | 0 | 0 | 6 |
| Cell adhesion impairing toxin | 0.300 | 0.667 | 0.414 | 18 |
| Viral exotoxin | 0 | 0 | 0 | 4 |
